# The right microbe-associated molecular patterns for effective recognition by plants

**DOI:** 10.3389/fmicb.2022.1019069

**Published:** 2022-09-26

**Authors:** Pengpeng Lü, Yi Liu, Xixi Yu, Chun-Lin Shi, Xiaokun Liu

**Affiliations:** ^1^Lushan Botanical Garden, Chinese Academy of Sciences, Jiujiang, Jiangxi, China; ^2^School of Life Sciences, Nanchang University, Nanchang, Jiangxi, China; ^3^ANGENOVO, Viken, Norway

**Keywords:** apoplast, microbe-associated molecular patterns, pattern recognition receptors, ligands, receptor-like kinases, receptor-like proteins

## Abstract

Plants are constantly exposed to diverse microbes and thus develop a sophisticated perceive system to distinguish non-self from self and identify non-self as friends or foes. Plants can detect microbes in apoplast *via* recognition of microbe-associated molecular patterns (MAMPs) by pattern recognition receptors (PRRs) on the cell surface to activate appropriate signaling in response to microbes. MAMPs are highly conserved but essential molecules of microbes and often buried in microbes’ complex structure. Mature MAMPs are released from microbes by invasion-induced hydrolytic enzymes in apoplast and accumulate in proximity of plasma membrane-localized PRRs to be perceived as ligands to activate downstream signaling. In response, microbes developed strategies to counteract these processing. Here, we review how the form, the concentration, and the size of mature MAMPs affect the PRR-mediated immune signaling. In particular, we describe some potential applications and explore potential open questions in the fields.

## Introduction

One of the most important evolutionary events in the history of life on earth is advent of the earliest land plants around 480 million years ago (Gehrig et al., 1996). During their establishment in terrestrial ecosystems, land plants have to adapt to an environment that houses a large variety of microbes such as fungi, oomycetes, bacteria, and viruses. From then on, if not earlier, plants and microbes have had continual interactions, influencing the evolution of both plants and microbes (Wang et al., 2010). To respond appropriately to such diverse microbes, plants have evolved abilities to distinguish self from non-self (Medzhitov and JanewayJr., 2002) and further identify non-self as friends or foes (Antolin-Llovera et al., 2014) through a sophisticated immune system. With these distinctions and identifications, plants can adapt to their environment by either activating immune responses to defend against pathogens or initiating symbiosis signaling to facilitate the accommodation of symbionts (Antolin-Llovera et al., 2014).

When microbes enter the apoplast through nature entries or wounded sites of plants, plants can detect microbes in apoplast *via* the recognition of Microbe/Pathogen-Associated Molecular Patterns (MAMPs/PAMPs; hereafter, referred to as MAMPs) by plant cell-surface localized Pattern Recognition Receptors (PRRs) (Macho and Zipfel, 2014) and activate appropriate downstream responses. The major classes of MAMPs include proteins, carbohydrates, lipids and nucleic acids, with common features: highly conserved structures, important functions for microbe survival and absence from the host plants (Nurnberger et al., 2004; Boutrot and Zipfel, 2017; Buscaill and van der Hoorn, 2021).

Plant PRRs are receptor-like kinases (RLKs) or receptor-like proteins (RLPs) that carry the extracellular leucine-rich repeat (LRR) or lysine motif (LysM) domain to confer the recognition of MAMPs (Buscaill and van der Hoorn, 2021). These MAMPs serve as the ligands for PRRs, while the binding specificity of ligand-receptor pairs is determined by both ligands (Felix et al., 1999) and PRR receptors (Gomez-Gomez and Boller, 2000). Such binding often induces the oligomerization of receptors (Liu et al., 2012) and their interaction with co-receptors, which is often required for the subsequent signal transduction (Shan et al., 2008; Liebrand et al., 2013). In addition to these typical MAMPs, some MAMPs (such as cerebroside) do not need a short ligand for recognizing (Koga et al., 1998). In general, effective physiological concentrations of ligands are required for their biological activities in plants (Albert et al., 2015).

The recognition of MAMPs by PRRs depends on both sides of MAMPs and PRRs; here we only focus on the MAMPs side to discuss how the form, the concentrations and the size of the MAMPs affect their recognitions and thus subsequent immune responses in host plants.

## Apoplast

In higher plants, nearly all cells are connected directly or indirectly by plasmodesmata into a single “organism” named as symplast and the space outside this symplast is known as apoplast, including the cell wall and the aqueous intercellular space (Erickson, 1986).

**FIGURE 1:**
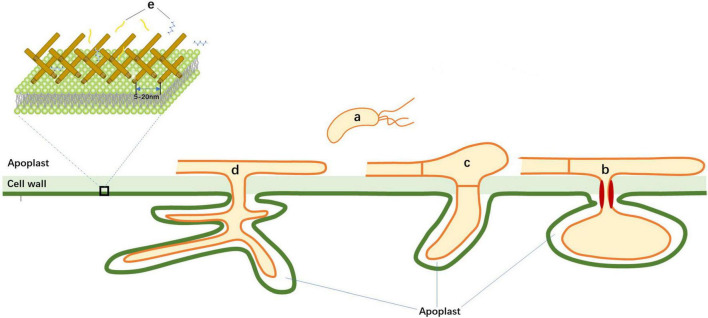
Schematic illustration of plant-microbe interface during invasion of pathogens. During invasion of microbes, bacterial, fungal, and oomycete pathogens colonize in apoplast space. Fungus/oomycete forms specialized structures known as the extrainvasive hyphal membrane (EIHM), the extrahaustorial membrane (EHM), or periarbuscular membrane (PAM). Mature microbe-associated molecular patterns (MAMPs) are derived from microbes and move through cell wall pores to be perceived by plasma-membrane localized Pattern Recognition Receptors (PRRs). **(a)** Bacterial pathogens; **(b)** Haustorial-forming fungal pathogens; **(c)** Non-haustorial-forming fungal pathogens; **(d)** Arbuscular mycorrhiza; and **(e)** Mature MAMPs.

In the scenario of plant-microbe interactions, the apoplast is the extracellular space in plant tissue that constitutes a source of nutrients and shelter for microbial inhabitants (Sattelmacher, 2001). Meanwhile, the apoplast is a hostile environment that contains hydrolytic enzymes and toxins for microbes (Sanchez-Vallet et al., 2015). Thus, the apoplast is defined as compartments of intracellular space beyond the plant plasma membrane, including the cell wall and structures that are formed during plant-microbe interactions (Figure 1). For example, fungal invasion forms a specialized structure known as appressorium. Further, invasive hyphae or haustoria are surrounded by a host-derived specialized membrane outside the invasive structure, known as the extrainvasive hyphal membrane (EIHM) (Kankanala et al., 2007), extrahaustorial membrane (EHM) (Karasov et al., 2017), or periarbuscular membrane (PAM) (Ivanov et al., 2019). The space between the microbe plasma membrane and the host extended membrane is also part of the apoplast (Wang et al., 2020; Figure 1).

Apoplast is a place where MAMPs matured from microbial complex. Apoplast constitutes a major battleground for plant-microbe interactions. Plants perceive microbial pattern *via* PRRs in apoplast to activate immune responses described as pattern-triggered immunity (PTI) (Saijo et al., 2018) while microbes develop extracellular strategies to avoid patterns recognition (Buscaill and van der Hoorn, 2021). Thus, the outcome of the apoplastic battle between plant and microbes will determine whether plants can stop invasion of microbes during early-stage infection.

## Forms of microbe-associated molecular patterns

Currently, the majority of known MAMPs are proteins, carbohydrates, lipids, and nucleic acids from bacteria, fungi, or oomycetes (see Table 1 and recent reviews for updated MAMPs and PRRs in Boutrot and Zipfel, 2017; Wang et al., 2020; Buscaill and van der Hoorn, 2021). Plant PRRs can perceive multiple MAMPs that are derived from flagellin, elongation factor Tu, peptidoglycans and lipopolysaccharides in bacteria, chitin in fungi, and Nep1-like Protein in oomycetes (Zipfel, 2014; Albert et al., 2015; Boutrot and Zipfel, 2017).

**TABLE 1:** Examples of some mature microbe-associated molecular patterns (MAMPs).

Precursor	MAMPs	Sequences	Concentration	Origin	PRR	Host	References
RaxX	RaxX21-sY	HVGGGDsYPPPGANPKHDPPPR	EC50 = 20 nM	*Xoo.*	XA21	Rice	Pruitt et al., 2015
Flagellin	Flg22	QRLSTGSRINSAKDDAAGLQIA	EC50 = 0.03 nM	*Pst.*	FLS2	Tomato	Felix et al., 1999
EF-Tu	elf18	ac-SKEKFERTKPHVNVGTIG	EC50 = 0.1 nM	*E. coli*	EFR	*Arabidopsis*	Kunze et al., 2004
XuP	Xup25	N.A	EC50 = 103 nM	*Pst.*	XPS1	*Arabidopsis*	Mott et al., 2016
CSP	csp22	AVGTVKWFNAEKGFGFITPDGG	EC50 = 0.1 nM	*Staphylococcus aureus*	CORE	*Tomato*	Felix and Boller, 2003
NLP	nlp20	AIMYSWYFPKDSPVTGLGHRHDWE	EC50 = 1.5 nM	*P. parasitica*	RLP23	*Arabidopsis*	Bohm et al., 2014
Chitin	(NAG)n	NAGn	EC50 = 0.1 nM	*Yeast cell wall*	CERK1	*Arabidopsis*	Felix et al., 1993
PGN	GlcNAC-MurNAC	(GlcNAC-MurNAC)n	Saturated 100 ug/ul	*S. aureus*	LYM1LYM3	*Arabidopsis*	Gust et al., 2007
LPS	LPS	LPS	Saturated 15 ug/ul	*Ps*	LORE	*Arabidopsis*	Ranf et al., 2015

The right form of MAMPs is necessary for the recognition by PRRs and effective activation of immune responses in plants. The PRRs can recognize and bind with the small epitopes of MAMPs as ligands. For a certain host PRR, the specificity and affinity of ligand binding to the PRR depends on the proper form of MAMP, such as its sequence variants and modifications (Nurnberger et al., 1994; Chinchilla et al., 2006).

### Microbe-associated molecular patterns variants

Microbe-associated molecular patterns are highly conserved patterns in microbes and maintain essential function for the microbe’s survival and therefore difficult to alter (Janeway, 1989; Medzhitov, 2007). Flg22 is one of the well-studied MAMPs derived from the bacterial flagellin (Felix et al., 1999; Zipfel et al., 2004). Flg22 is a highly conserved 22-amino acid of the *N*-terminal domain of bacterial flagellin (Table 1; Felix et al., 1999; Zipfel et al., 2004), and can be recognized by Flagellin Sensing 2 (FLS2) (Gomez-Gomez and Boller, 2000) to trigger immune responses.

However, some microbes can avoid the detection by PRRs through mutating sequence of MAMPs. Mutations in flg22 that are not recognized by FLS2 was reported in some flagellated bacteria (Cheng et al., 2021). An α-proteobacterium Agrobacterium carrying a flg22 variant does not trigger FLS2-mediated immune responses (Felix et al., 1999). With different versions of the flg22, some strains of the bacterial *Ralstonia solanacearum* and *Xanthomonas campestris* pv. *campestris* render them undetectable for FLS2-mediated immune system in host plants (Pfund et al., 2004; Sun et al., 2006). Likely, *Xanthomonas oryzae* pv. *oryzae* (*Xoo*) and pv. *oryzicola* (*Xoc*) can escape FLS2-mediated detection in rice due to substitutions in flg22 sequence (Wang et al., 2015). Further, *X. arboricola* pv. *juglandis* with the conserved flg22 sequence is non-pathogenic but strains with mutation within the flg22 sequence that can evade the recognition by FLS2 is pathogenic (Cesbron et al., 2015). The specific recognition of MAMPs relies on the interaction between the extracellular domain of PRRs and MAMPs, while the induction of immune responses in plant cells is determined by the activation of the intracellular domain of PRRs, the kinase domain. Interestingly, chimeric FLS2 receptors with partially swapped extracellular domains between the AtFLS2 and SlFLS2, could recognize both flg22 in *Arabidopsis* and flg15 in tomato (Mueller et al., 2012). However, whether a single native/chimeric PRR could recognize various MAMPs and mediate broad pathogen resistances in plants still requires further studies.

Besides flg22, elf18 is another well-studied MAMPs which contain the first 18 amino acids of *N*-terminus of the highly conserved bacterial protein Elongation factor (Table 1; Kunze et al., 2004), and can be recognized by the EF-Tu Receptor (EFR) to activate defense responses (Zipfel et al., 2006). Mutations in elf18 can also affect the immune activity in plants. The elf18 variants of *X. campestris* pv. *campestris* (*Xcc*) and *Pseudomonas syringae* pv. *tomato* DC3000 (*Pto*DC3000) trigger only 0.8–3.2% of the immune activity in comparison to that from *Agrobacterium*, *Ralstonia*, and other *Xanthomonas* and *Pseudomonas* strains (Lacombe et al., 2010).

Another example of MAMP with sequence variant is RaxX, a highly conserved protein in many pathogenic *Xanthomonas* species. RaxX is recognized by the rice receptor kinase XA21 to confer resistance to most strains of *Xoo* (Table 1; Wang et al., 1996; Pruitt et al., 2015; Luu et al., 2019). However, *Xoo* isolates with substitutions of the Y41 residue in RaxX can avoid XA21-mediated defense (Pruitt et al., 2015).

### Microbe-associated molecular patterns modifications

Modification of the MAMPs is another strategy to render them undetectable by plant PRRs and avoid host resistance.

Chitin is a structural unit of the fungal cell walls (Table 1) and chitin fragments are recognized as MAMPs by cell surface receptor Chitin Elicitor Receptor Kinase1 (CERK1) and Chitin Elicitor-Binding Protein (CEBiP) in plants (Kaku et al., 2006; Miya et al., 2007; Wan et al., 2008). Deacetylating chitin into chitosan prevents the recognition of chitin by receptors to evade host immunity (Vander et al., 1998). For example, fungal pathogens *Verticillium dahliae* and *Fusarium oxysporum* f. sp. *vasinfectum* secrete polysaccharide deacetylase1 (PDA1) to deacetylate chitin to prevent chitin-triggered immunity in cotton (Gao et al., 2019). The wheat stripe rust fungus *Puccinia striiformis* f. sp. *tritici*, the broad bean rust fungus *Uromyces fabae*, and the maize anthracnose fungus *Colletotrichum graminicola* also replace chitin with chitosan in their hyphae to avoid immunity (El Gueddari et al., 2002). In addition, it was reported that chitin deacetylases in the endophytic fungus *Pestalotiopsis* sp. are induced to avoid the recognition by plants (Cord-Landwehr et al., 2016).

Lipopolysaccharides (LPSs) are the major component of the outer membrane of Gram-negative bacteria (Table 1). LPS consists of lipid A and an oligosaccharide core that carries an O-polysaccharide (OPS) (Dow et al., 2000). LPS can be sensed by plant receptor-like kinase LORE and induce immune responses (Ranf et al., 2015). It was found that the pathogenic bacterium *Xylella fastidiosa* with an O-antigen chain delays the recognition by host plants while mutants of O-antigens strains induce faster immune responses, suggesting that glycan modification of LPS may affect plant immunogenic recognition (Rapicavoli et al., 2018).

There is an ongoing arm race between the immunorecognition by PRRs in host plants and the evasion of immunorecognition by pathogens. The race drives adaptive evolution of MAMPs in form to evade immunorecognition, though MAMPs are highly conserved patterns which are essential for microbes. Adaptive evolutions of MAMPs in sequences and modifications render them undetectable by PRRs and evade immunorecognition; nine different extracellular strategies have been recently summarized on how microbes avoid recognition by the host (Buscaill and van der Hoorn, 2021). In response to this, plants also evolve new versions of PRRs which can recognize these new forms of the MAMPs (Mueller et al., 2012).

In summary, immunogenic recognition of MAMPs by plant PRRs, which is determined by the specificity and affinity of ligand binding to PRRs, requires the proper form of MAMPs with right sequences and modifications.

## Concentration of microbe-associated molecular patterns

Plants recognize MAMPs in a dose-dependent manner (Albert et al., 2015), and thus the effective activation of immune responses requires enough physiological concentration of the proper MAMPs at the infection site. The exact physiological concentration of MAMPs is difficult to measure due to technique limits. However, *in vitro* assays showed that the activity of MAMPs ranges from pM to nM (Table 1). We reason the exact physiological concentration of MAMPs on site should be lower or equal to pM level since not 100% of exogenous MAMPs could reach to the place where PRRs are. Upon infection, the concentration of MAMPs on site is dynamic and depends mainly on the production of MAMPs. It is generally known MAMPs are produced through two ways: biosynthesis and host hydrolyzation.

### Generation of microbe-associated molecular patterns through biosynthesis

During infection, large amounts of peptidoglycan (PGN) building blocks are biosynthesized and some of these blocks are steadily shed into extracytoplasmic space. For instance, *Bacillus subtilis* released about 50% of its PGN in one generation during growth (Goodell and Schwarz, 1985). It has also been found that flagellin monomers are released into the supernatant of *Pseudomonas aeruginosa* cultures (Bardoel et al., 2011). Likewise, LPSs dropped into liquid culture when *Escherichia coli* grows *in vitro* (Mackowiak, 1984). Upon infection, these building blocks fallen into space surrounding cells and can serve as MAMPs for the activation of PTI signaling. For instance, culture supernatants from *Bacillus* sp. were shown to cause immune responses through the activation of nucleotide-binding oligomerization domain-containing protein (NOD), a signaling cascade in response to mostly either meso-diaminopimelic acid (mDAP) containing cell wall peptides (Fujimoto and Fukase, 2011). *Pseudomonas aeruginosa aprA* mutants induced an activation of TLR5 signaling *via* shedding monomeric flagellin into their environment (Bardoel et al., 2011). Similarly, growing fungal cells also shed chitin into the environment (Bueter et al., 2013).

To reduce the immune responses in plants, microbes downregulate the biosynthesis of MAMPs to reduce the concentrations of MAMPs on site. It was found that the biosynthesis of flagella in *Pseudomonas* is downregulated by the second messenger cyclic-di-GMP (cdG) (Hickman and Harwood, 2008). Increased cdG levels in the plant pathogen *P. syringae*, the plant opportunist *P. aeruginosa* and the plant commensal *Pseudomonas fluorescens* reduce flagellin levels, and thus reduce flg22 concentration on site, which contributes to the evasion of FLS2-mediated immune response in *Nicotiana benthamiana* and Arabidopsis (Pfeilmeier et al., 2016). Similarly, the maize fungal pathogen *C. graminicola* downregulates the expression of genes encoding KRE5 and KRE6, which are key enzymes for the biosynthesis of β-glucan (Oliveira-Garcia and Deising, 2016).

### Generation of microbe-associated molecular patterns through host hydrolytic enzymes

On the other hand, host hydrolytic activities establish decomposition of MAMPs precursors in apoplast to generate soluble ligands for PRRs. Plant apoplast contains hundreds of glycosidases, proteases, and other hydrolases (Grosse-Holz et al., 2018). In general, these hydrolases in the apoplast are not considered directly detrimental to pathogen growth, but are presumed to release pathogen MAMPs, which in turn trigger downstream defense responses. For example, fugal pathogens often induce plant chitinases to target the fungal cell walls, releasing chitin as MAMPs (Sanchez-Vallet et al., 2015). Upon bacterial infection, plants can produce a metazoan lysozyme-like hydrolase (LYS1), which releases soluble PGN from insoluble bacterial cell walls for triggering plant immunity (Liu et al., 2014). Similarly, immunogenic flagellin peptides are released from flagellin by host proteases with glycosidases to trigger immunity (Buscaill et al., 2019).

In response to host hydrolyzation, microbes can secret proteins or glycans to protect MAMPs precursors from hydrolases. One method is to secret proteins that cover the hydrolytic sites in MAMP precursors. Tomato leaf mold fungus produced Avr4 binds specifically to chitin in the fungal cell walls to protect it from plant chitinases (van den Burg et al., 2006). Similarly, xylem-invading fungus *Verticillium nonalfalfae* prevents chitin from hydrolysis by secreting VnaChtBP that binds chitin and suppresses chitin-induced immunity (Volk et al., 2019). Interestingly, the fungal vascular wilt pathogen *V. dahliae* strain VdLs17 could secret a lineage-specific LysM effector, Vd2LysM, which mimics the host PRR to bind chitin and suppresses chitin-induced immune responses (Akcapinar et al., 2015).

Glycosylation of MAMP precursors is another approach employed by microbes to evade host recognitions. For instance, glycosylation of bacterial flagellin and fungal chitin suppress the release of MAMPs. O-glycosylation of flagellin is observed in bacterial pathogens such as *Xanthomonas*, *Pseudomonas, Burkholderia*, *Dickeya*, *Erwinia*, *Pantoea*, and *Pectobacterium* (Taguchi et al., 2010; Ichinose et al., 2013; De Maayer and Cowan, 2016), presumably preventing the hydrolytic release of the flagellin MAMP (Buscaill and van der Hoorn, 2021). While mutants of the flagellin glycosyltransferase in *P. syringae* pv. *tabaci 6605*, *P. syringae* pv. *glycinea* race 4 and *X. campestris* pv. *campestris Xca* showed less virulent to their host plants (Takeuchi et al., 2003; Taguchi et al., 2006; Ichinose et al., 2013).

In bacterial pathogens *Acidovorax avenae* which cause rice leaf blight, flagellin from the N1141 strain but not K1 strain induces immune responses. The flagellin in two strains are identical in sequences but different in glycosylation pattern such as a 1,600 Da O-glucan for N1141 while a 1,600-Da one for K1 (Hirai et al., 2011).

Glycosylation of fungal cell walls could also prevent the release of chitin from cell wall. For instance, rice pathogens *Magnaporthe oryzae*, *Cochliobolus miyabeanus*, and *Rhizoctonia solani* accumulate α-1,3-glucan on the surface of infectious hyphae (Fujikawa et al., 2009, 2012). While fungal mutants with reduced α-1,3-glucan levels showed less virulence, suggesting that α-1,3-glucan may prevent chitin release from cell wall (Fujikawa et al., 2012).

In summary, the physiological concentration of the proper MAMPs, which is required for activation of effective immune responses at the infection site, is dependent on both biosynthesis of MAMPs by microbes and hydrolytic release of MAMPs by plants.

## Size of microbe-associated molecular patterns

In apoplast, mature MAMPs accumulate in the proximity of the plasma membrane-localized PRRs through biosynthesis (Mackowiak, 1984; Goodell and Schwarz, 1985; Bardoel et al., 2011) or host hydrolytic enzymes (Guimil et al., 2005; Liu et al., 2014; Buscaill et al., 2019). The epitopes of MAMPs serve as ligands for PRR recognition and the immunogenic units of MAMPs are often buried in the MAMP precursors that are essential structures of microbes such as cell wall (Kaku et al., 2006; Miya et al., 2007) and flagella (Felix et al., 1999; Zipfel et al., 2004).

For the effective MAMP-PRR recognition, firstly MAMP molecules must be released as soluble fragments from precursors by host hydrolytic enzymes. Then these fragments need to move cross the host cell wall matrix before being recognized as ligands by plant plasma membrane-localized PRRs (Figure 1).

**FIGURE 2:**
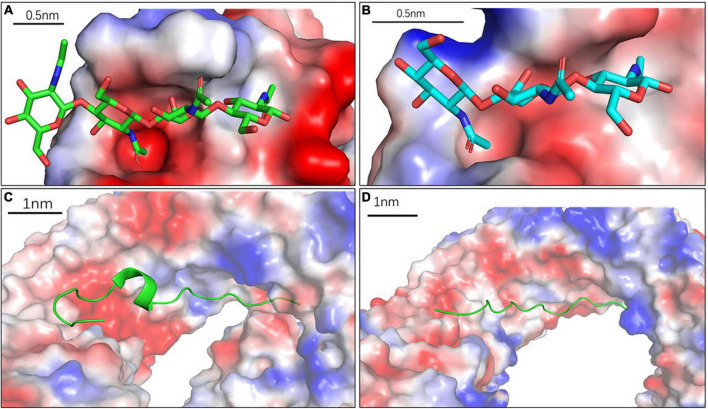
The predicted size of the mature microbe-associated molecular patterns (MAMPs) from crystal structure of Pattern Recognition Receptor (PRR)-ligand pairs. **(A)** Chitin in atCERK1, (NAG)4 = 2.07 nm; **(B)** Chitin in OsCEBiP, (NAG)3 = 1.54 nm; **(C)** flg22, flg22 in Flagellin Sensing 2 (FLS2) length of flg22 = 4.06 nm; **(D)** atPEP1 in atPEPR, length of atPep1 = 4.4 nm.

Plant cell walls compose of cross-linked polysaccharides with pores, ranged from 5 to 20 nm in size (Wang et al., 2016; Cunningham et al., 2018), suggesting that any MAMPs with size greater than these pores could be limited in their ability to pass through the cell wall matrix. Based on these criteria, the size of the mature MAMPs should be less than the pore size of cell wall matrix (5 nm). The immunogenic fragments of chitin, (NAG)7–8, can induce the highest immune activity in plants (Zipfel, 2008). Consistent with this, the length of (NAG)8 is around 4.14 and 4.08 nm (Figure 2), based on the crystal structures of the chitin receptors atCERK1 and OsCEBiP, respectively (Liu et al., 2012, 2016). Likewise, the length of flg22 from flagellin is 4.6 nm (Figure 2; Sun et al., 2013). Further, the length of atPep1 is 4.4 nm (Tang et al., 2015). As expected, these mature MAMPs have a size of less than 5 nm, which allows them to move through the cell wall matrix before specifical binding to their PM-localized PRRs.

However, extensive hydrolysis of the immunogenic MAMPs often leads to the loss of immunogenic activity (Liu et al., 2012, 2014). For instance, the heptamer and octamer of chitin fragments showed the highest immunogenic activity (Zipfel, 2008), but little (Petutschnig et al., 2010) or no activity (Zhang et al., 2002) was observed for tetramers and pentamers of chitin. Similarly, extensive digest of PGN into small fragments appears to abolish its immunogenic activity (Liu et al., 2014). Presumably, the minimum length of immunogenic fragments is required for the oligomerization of PRRs or interactions between PRRs and co-receptors, which is often necessary for effectively activating immune signaling (Liu et al., 2012). Furthermore, these small fragments by extensive hydrolysis of the immunogenic MAMPs may compete the immunogenic ligands for PRR binding, and therefore lead to the desensitization of the PRR-mediated perception (Felix et al., 1998).

In summary, the size of proper MAMPs with immunogenic activity falls in such a range that allow themselves to move through cell wall pores, as well as to bind with PRRs and initiate effective immune signaling.

## Conclusion and perspectives

During plant-microbe co-evolution, plant developed a sophisticated system to detect pathogens in apoplast and activate appropriate signaling in response to microbial invasions. Instead of direct perceiving entire microbes, plant only recognizes microbial signatures (MAMPs) by the cell surface receptors (PRRs) to initiate immune responses. Here, we discussed the form, the concentration, and the size of the mature MAMPs for effective activation of immune signaling in plants.

In plant apoplast, the cell wall matrix forms a barrier between apoplastic microbes and plant plasma membrane-localized PRRs (Figure 1). On one hand, the cell wall acts as a physical barrier to prevent microbes from contacting directly with plasma membrane. On the other hand, plant cell needs to overcome this distance barrier to directly detect the invasion of microbes to initiate immune responses. Investigation of the size range of immunogenic MAMPs will aid to understand the recognition and activation mechanisms of PTI, and develop new effective disease resistance strategies in plants. Furthermore, MAMPs can trigger plants to switch to a primed state of enhanced defense known as defense priming, which can also be induced by synthetic chemicals (Conrath et al., 2015). Knowledge of the size range of immunogenic MAMPs helps to design synthetic molecules as efficient priming inducers.

Recognition of MAMPs by PRRs triggers PTI which is locally and transiently, thus the fine-tuning signaling is required for plants in responds to such diverse microbes. We described that the PAMPs can be generated by both microbe biosynthesis and plant hydrolytic cleavage, while the concentration of PAMPs is temporally dynamic at the infection site of plant. In a temporal resolution, invasion of pathogens in apoplast initially generate low levels of MAMPs, which can be detected immediately by PRRs to induce signaling, including the upregulation of hydrolytic enzymes in apoplast (Liu et al., 2014). Consequently, increased levels of hydrolytic enzymes in apoplast release more MAMPs from pathogens and the elevated concentration of MAMPs then trigger stronger PTI. When pathogens are inhibited by immune responses and decreased in apoplast, MAMP precursors decrease subsequently. As a result, high levels of apoplastic hydrolytic enzymes over-digest the MAMPs into small fragments, leading to the low concentration of immunogenic MAMPs. Small fragments can further compete with immunogenic MAMPs for PRR binding and reduce PTI, which in turn prevents the upregulation of hydrolytic enzymes. Thus, the temporal dynamic of MAMPs enables plant to respond to the invasion of pathogens in an appropriate manner. However, it is unknown if there is a spatial regulation of MAMPs in apoplast since there is no any separated space in apoplast.

In conclusion, upstreaming events of recognition of MAMPs by PRRs are exciting research fields. However, there are still many holding secrets in how MAMPs mature from precursors, further understandings of these apoplastic events will ultimately lead to novel strategies to enhance pathogen resistances in plants.

## Author contributions

XL: writing–original draft and supervision. PL, YL, XY, C-LS, and XL: conceptualization, writing–review and editing, and approved the submitted version.
